# Ether in the developing world: rethinking an abandoned agent

**DOI:** 10.1186/s12871-015-0128-3

**Published:** 2015-10-16

**Authors:** Connie Y. Chang, Elisabeth Goldstein, Nitin Agarwal, Kenneth G. Swan

**Affiliations:** 1 Department of Anesthesia, Rutgers New Jersey Medical School, Newark, NJ 07101-1709 USA; 2 Department of Surgery, Rutgers New Jersey Medical School, Newark, NJ USA; 3 Department of Neurological Surgery, University of Pittsburgh Medical Center, Pittsburgh, PA USA

**Keywords:** Ether, Halothane, William T.G. Morton, Charles A. Jackson, Oliver Wendell Holmes Sr, Dr. John Warren

## Abstract

**Background:**

The first true demonstration of ether as an inhalation anesthetic was on October 16, 1846 by William T.G. Morton, a Boston dentist. Ether has been replaced completely by newer inhalation agents and open drop delivery systems have been exchanged for complicated vaporizers and monitoring systems. Anesthesia in the developing world, however, where lack of financial stability has halted the development of the field, still closely resembles primitive anesthetics.

**Discussion:**

In areas where resources are scarce, patients are often not given supplemental intraoperative analgesia. While halothane provides little analgesia, ether provides excellent intra-operative pain control that can extend for several hours into the postoperative period. An important barrier to the widespread use of ether is availability. With decreasing demand, production of the inexpensive inhalation agent has fallen.

**Summary:**

Ether is inexpensive to manufacture, and encouraging increased production at a local level would help developing nations to cut costs and become more self-sufficient.

## Background

First world anesthesiology delivers excellent care in a safe, reliable environment. Safe medications, advanced vaporizers and complex intra-operative monitoring are standard in western operating rooms. Anesthesiology in the developing world, however, is vastly different from its western counterpart. In countries where health systems are plagued by diseases such as HIV/AIDS and malaria, anesthesiology is seen as a low priority and lacks the voice to demand access to better resources. In Uganda, for example, only 23 % of anesthetists had the minimal requirements for the safe provision of anesthesia to an adult. An anesthetist is an anesthesia provider, often a nurse or someone other than a physician. The items that this study found to be most frequently unavailable were a pulse oximeter, a tilting operating table, an oxygen source, and appropriately sized endotracheal tubes. Furthermore, the authors also found that running water was not always present and intravenous fluids were not readily available. Shockingly, only 16 % of government hospitals and 50 % of mission hospitals were able to deliver safe anesthesia for adults [1]. It is in this context, that a case is made for the reexamination and promotion of ether as the primary inhalation general anesthetic of the developing world.

## Discussion

### Historical perspectives

The discovery of ether for the use as an anesthetic was in 1846 which marked the birth of a modern age in anesthesiology. Although its use has been abandoned in the developed world, ether was safely and effectively used as an inhalation anesthetic for over one hundred years. Prior to the middle of the 19^th^ century and the discovery of ether, surgery was a rare and gruesome procedure. One of the most common operations was extremity amputation. There the surgeon used saws and knives to remove an appendage and scalding irons to cauterize the wound. Before the discovery of anesthesia, the sound of patients thrashing and screaming vibrated throughout the operating rooms [2]. They resorted to a variety of methods to control the patient’s pain including alcohol, opiates, ice, and various distractions.

Ether (diethyl ether) was first prepared in 1540 by Valerius Cordus, a Prussian botanist. Cordus produced the compound, known as “sulfuric ether” at the time, by distilling sulfuric acid (oil of vitriol) with fortified wine to make an “oleum vitrioli dulce” (sweet oil of vitriol) [3]. Despite this early synthesis, ether was rarely used over the next three decades. In fact, it’s only routine consumption was as a recreational drug among poor Britians who sometimes drank an ounce of ether when traditional alcohol was not available. American students adopted a variation of this practice in the “ether frolics” of the early 1800s to achieve a feeling of euphoria. Participants would hold ether soaked towels to their faces until losing consciousness.

Ether was first made use of as a general anesthetic by Dr. Crawford Williamson Long on March 30, 1842. Long was a physician and pharmacist who learned about ether while studying medicine in college. In 1842, Long removed a tumor from the neck of a patient who was under the effects of ether anesthesia. Unfortunately, the successful and unprecedented use of anesthesia during surgery was not credited to Long due to his laxity in publishing the results of the surgery until several years later.

The first true demonstration of ether as an inhalation anesthetic was on October 16, 1846 by William T.G. Morton, a Boston dentist. He discovered the anesthetic properties of ether in his search to provide patients with relief from painful dental procedures [4]. Prior to 1846, anesthesia was not used during surgical procedures and patients often avoided surgical intervention at all cost. Additionally, prior to the monumental events of 1846, Morton attended a public demonstration of the anesthetic properties of nitrous oxide given by Horace Wells. Wells, also a dentist, administered nitrous oxide to a patient and then extracted a wisdom tooth. Unfortunately, the patient did not appear to be fully anesthetized and the public deemed the demonstration to be a failure.

The exhibition furthered Morton’s interest in inhalation anesthetics and led him to consult a professor of chemistry at Harvard Medical School, Charles A. Jackson [4]. Jackson recommended a trial of sulfuric ether as an alternative to nitrous oxide, commonly known as “laughing gas”. Morton began experimenting with the ether and learned that a drop on the skin produced local analgesia. Further investigation with inhaled ether revealed that the agent was more versatile than inhaled nitrous oxide. As opposed to nitrous oxide, bottles of ether could be transported easily and the volatility of the drug permitted effective inhalation. In addition, the concentration of the drug required for surgical anesthesia was so low that the patients did not become hypoxic when breathing ether vaporized in air which is an advantage compared to nitrous oxide. Morton, who had entrepreneurial aspirations, quickly became convinced that ether was a suitable surgery anesthetic for hospital surgery and knew the significance of ether as a surgical anesthetic. He also knew that since ether has been available for centuries, it could not be patented, so Morton added some odor-masking impurities to ether, and called the concoction Letheon.

As a result of Morton’s “success”, he began to promote the use of ether in his dental practice. His success also prompted a demonstration at Harvard Medical School. On October 16, 1846, Morton publicly administered inhaled ether to a patient at Massachusetts General Hospital which took place in an amphitheater now known as the “Ether Dome”. The patient was successfully anesthetized and the surgeon, Dr. John Warren, removed a tumor from Mr. Gilbert Abbot’s neck. The patient had muttered as if in a semi-conscious state during the operation and after the surgery was complete, he stated that the pain was considerable, though mitigated. On the following day, the vapor was administered to another patient with complete success [4]. The public demonstration of ether anesthesia signaled the birth of the field of anesthesiology as a specialty. Ether anesthesia was adopted rapidly around the world and surgical anesthesia and analgesia became an accepted standard in surgical care.

Morton kept the true composition of Letheon a secret with the hopes of becoming wealthy. However, it was not long before surgeons recognized the smell of Letheon and associated it with ether. Morton attempted to fight the widespread use of ether which even included petitioning Congress for two decades to award him as the discoverer of ether. Nonetheless, Congress was knowledgeable of ether’s ancient origins and no monetary reward was given to Morton.

Oliver Wendell Holmes Sr. created the term anesthesia that was derived from the Greek word *anaisthēsia* meaning “lack of sensation” [5]. Holmes was a professor of anatomy and physiology at Harvard and had rejoiced saying that “The knife is searching for disease, the pulleys are dragging back dislocated limbs, nature herself is working out the primal curse which doomed the tenderest of her creatures to the sharpest of her trials, but the fierce extremity of suffering has been steeped in the waters of forgetfulness, and the deepest furrow in the knotted brow of agony has been smoothed forever [6].”

Ether was safe, easy to use, and remained the standard general anesthetic until the 1960s when the fluorinated hydrocarbons (halothane, enflurane, isofluorane and sevoflurane) came into common use. Although these newer agents reduced the postoperative nausea, vomiting and flammability that were problematic with ether, they were expensive to produce and included a host of undesirable side effects.

Anesthetics used today are almost unrecognizable from anesthetics used in the late 1800s. Ether has been replaced completely by newer inhalation agents and open drop delivery systems have been exchanged for complicated vaporizers and monitoring systems. Anesthesia in the developing world, however, where lack of financial stability has halted the development of the field, still closely resembles primitive anesthetics.

### Anesthesia in undeveloped countries

Shortages of personnel who are adequately trained to administer anesthetics, drugs, and equipment are commonplace in third world countries. Operating room conditions are primitive by U.S. standards [7]. Pulse oximeters, tilting operating tables and appropriately sized endotracheal tubes are often unavailable. A functional oxygen source, running water and electricity are often unreliable. Furthermore, there are an inadequate number of anesthesiologists in third world nations and anesthesia is also usually delivered by non-physician anesthetists and nurses under the direction of a surgeon. These providers typically have minimal or no formal training in medicine and usually even less training in anesthesiology [1, 8]. A recent study in Uganda, for example, found that among 91 anesthesia providers, 85 % attended training courses of only 1 year to 19 months duration. The same study found that there are only 13 anesthesiologists and 330 non physician anesthetists for the entire Ugandan population of 27 million. By comparison, there are approximately 12,000 anesthesiologists for a population of 60 million in the United Kingdom [1].

In an attempt to provide anesthesia, developing countries have had to make cost saving adjustments. “One time use” items such as endotracheal tubes are reused and recycled, often becoming unsafe because of balloon rupture after a few uses [9]. Expensive anesthetic vaporizers and circle systems, the mainstay of western anesthesiology have been largely abandoned. These devices are not only expensive, but they are also costly to maintain. Service, parts, and appropriate safety training eparts are rarely available.. In addition, these vaporizers are dependent on a continuous supply of oxygen, which is not reliably available in developing nations.

Consequently, “draw-over anesthesia” is extensively utilized. In draw-over anesthesia, the carrier gas (atmospheric air) is drawn over the volatile liquid (anesthetic agent) by the patients’ respiratory efforts. Drawover systems are simple to assemble and use. Most importantly, drawover technique is safe for patients.

Anesthetic drugs, common in western anesthesiology, are rarely available in developing nations. Sevofluorane, desfluorane and isofluorane, the fluorinated hydrocarbons that are the major inhalation agents of the west, are unavailable in developing countries. These agents are both expensive and require extensive equipment for delivery. Instead, halothane is the most widely used volatile agent in developing nations [8]. Ether is also used, but its use is limited due to decreased availability and flammability. Substituting ether for halothane in developing nations, most importantly in those already using drawover anesthesia, could both save money and improve the safety of surgical anesthesia.

### Ether versus halothane

Although abandoned in western anesthesia, ether has long been known as a relatively safe and inexpensive anesthetic. Halothane costs approximately U.S. $140 per liter (U.S. $0.14 per ml). In one study, Eltingham, using a Glostavent Anesthetic Machine with halothane as the sole agent, found that the mean use of halothane was 16 ml per hour. This results in a cost of U.S. $2.24 per hour of use. While this cost is relatively minor by western standards (approximately a tenth of the price of Sevoflurane), it is still a considerable portion of the small budget of anesthesiology departments in developing nations. In a study conducted in 1994 in Malawi, halothane accounted for a quarter of the entire anesthesiology department budget [8].

In contrast, ether costs a mere U.S. $10 per liter (approximately U.S. $0.01 per ml). Using the same calculations as above, this would lead to a cost of U.S. $0.16 per hour of use (a U.S. $2.08 per hour savings compared to halothane). The cost difference could save struggling anesthesiology departments thousands of dollars each year.

Hospitals located in rural areas could also save money by increasing the use and production of ether. While halothane is relatively expensive to produce and must be manufactured in large factories and then transported to rural health care centers, ether can be easily and cheaply produced from ethanol at a local level. Purchasing locally manufactured anesthetic ether could have significant effects by decreasing cost, supporting local economies, and making rural hospitals in developing nations more self-sufficient.

Both halothane and ether can be administered easily using drawover anesthetic techniques. Halothane, however, is a relatively unsafe agent to use without intra-operative monitoring. As mentioned earlier, developing nations rarely have either a reliable oxygen source or electricity. As a result, intra-operative monitoring and intervention with supplemental oxygen are not possible. Halothane depresses both the ventilatory drive and cardiac output making supplemental oxygen and cardiac monitoring crucial. Ether, on the other hand, acts as a sympathomimetic agonist, stimulating cardiac output, respiratory rate, and causing bronchodilation. While not ideal, it is therefore safe to use ether when supplemental oxygen, endotracheal intubation and cardiac monitoring aren’t available.

Finally, the side effects of ether anesthesia are relatively minor when compared with those of halothane. Halothane hepatitis is a well-documented toxicity in patients exposed to the drug. Although relatively rare (affecting only 1/10,000 patients), halothane hepatitis has a mortality rate of 50 % [10]. In addition, halothane has been known to cause cardiac arrhythmias as well as fatal bradycardia. Due to the concerns surrounding its hepatotoxicity, halothane has been primarily eliminated for use in adults in the United States and many other countries. Halothane was eventually replaced by safer newer volatile anesthetics. However, in countries with different medico legal climates, halothane still plays a vital role due to its relatively low cost. For example, halothane is still being used as the main anesthetic in more than 80 % of the hospitals in Iran. As a result, increasing numbers of halothane hepatitis is being reported in Iran and among other countries still using halothane [11]. Ether, on the other hand, is a relatively safe agent with its main side effect being postoperative nausea and vomiting.

The major problem with ether is its extreme flammability, especially in the presence of oxygen. Ether is extremely volatile and has a low boiling point, two characteristics which make it prone to explosion. The number of surgical fires in 1960 (when ether was the primary inhalation anesthetic used in the United States), was reported at one in approximately 100,000 anesthesia [12]. Therefore, the number of fires caused by ether is therefore relatively small.

There are relatively easy to implement precautions that operating rooms in developing countries can mandate to combat the ether’s flammability. First, open flames, such as those provided by alcohol lamps, Bunsen burners, matches, and smoking must be prohibited in rooms where anesthetics are either administered or present. Next, the use of incandescent or high frequency cautery or coagulation within a distance of two feet from the mouth of a patient receiving flammable anesthetics must be prohibited unless a rubber sheet and wet drapes are properly applied. Last, ether should be stored in original cans or dark glass bottles to avoid explosions due to the effect of sunlight [12].

### Barriers to widespread use

While halothane is currently the most widely used volatile agent in the developing world, ether still has a place in some counties. Unfortunately, even this use is threatened by decreased availability of ether and lack of medical provider education in ether anesthesia. Lack of funding to anesthesia departments in developing nations has led many budding anesthesiologists to seek training in wealthier countries. Unfortunately, education in ether anesthesia and drawover techniques has completely vanished from the curriculum of the first world. This leads to medical migration; anesthesiologists from developing countries who are trained abroad often remain in the countries where they are educated after attaining their training. In addition, volunteer practitioners who travel to developing nations from the west are unfamiliar with ether and drawover techniques and often find themselves unequipped to deal with the realities of delivering anesthesia in the developing world. Anesthetic ether has also become less available in recent years. Falling demand for the inexpensive agent in developed nations has led many manufacturers to claim a lack of profitability and to halt production [1].

## Summary

Diethyl ether anesthesia deserves to be reconsidered for widespread use in the developing world. In countries that lack the resources to fund anesthesiology development, ether could greatly improve the safety and economics of anesthesia practices.

Developing nations rarely have the personnel and equipment to provide safe anesthesia. Anesthesia is most commonly delivered by non-physicians who have little or no formal training. Cardiac monitors, pulse-oximeters, supplemental oxygen and endotracheal intubation are rarely available [1], and anesthesia is delivered using drawover techniques. Halothane, the most common inhalation anesthetic in the developing world, is a potent agent and, without monitoring equipment and trained providers, can lead to significant patient morbidity and mortality. Ether, on the other hand is nontoxic to the cardiovascular system and it does not depress respiratory activity. It is safe to use by anesthetists who have not received formal training and without complicated monitoring, supplemental oxygen and endotracheal intubation. Ether has the added benefit of providing surgical analgesia. In areas where resources are scarce, patients are often not given supplemental intraoperative analgesia. While halothane provides little analgesia, ether provides excellent intra-operative pain control that can extend for several hours into the postoperative period.

Finally, ether is inexpensive and would provide significant cost savings in countries where halothane represents a major portion of anesthesiology department budgets. While halothane costs approximately U.S. $140 per liter (U.S. $0.14 per ml), anesthetic ether can be purchased for as little as U.S. $10 per liter (U.S. $0.01 per mL). Assuming a mean use of 16 ml per hour, this leads to a cost savings of approximately U.S. $2.24 per hour of use. Promoting ether anesthesia for widespread use in developing countries could lead to enormous savings, which could be channeled into improving training, equipment and the overall conditions of anesthesia services.

While it has been shown that ether anesthesia is safe and inexpensive, there are significant barriers to its widespread use. Anesthesiologists in developed nations are completely unfamiliar with the agents and the drawover techniques required for its delivery. As many anesthesiologists from developing countries are trained in the west, the lack of education in ether anesthesia and relevant techniques leaves them unprepared to practice in their home countries. Similarly, volunteer anesthesiologists from developed nations seeking to help under resourced hospitals find themselves lacking in the skills necessary to deliver anesthesia in a new environment.

The solution to this problem is twofold. First, education in drawover anesthesia, using ether as the primary inhalation agent, should be reinstated as part of the educational curriculum in developed countries. Encouraging familiarity with these anesthetic techniques would allow all anesthesiologists to be proficient in the delivery of anesthesia in developing countries. Second, academic institutions in developed nations should be encouraged to establish joint training program in developing countries. Such programs would benefit both sponsor institutions and the under resourced areas where they are located. Anesthesiologists in developed countries would receive a broader education in anesthetic technique and those in developing countries would receive proficient training in their home countries; thereby halting medical migration.

The last barrier to the widespread use of ether is availability. With decreasing demand, production of the inexpensive inhalation agent has fallen. Ether is inexpensive to manufacture, and encouraging increased production at a local level would help developing nations to cut costs and become more self-sufficient.

The widespread use of ether anesthesia in developing countries would improve patient safety, cut costs and help struggling health care systems become more self-sufficient. In 2007, the British Medical Journal asked subscribers to name the most-significant medical developments since 1840, and unsurprisingly, anesthesia was among the top three which also included antibiotics and modern sanitation.

## References

[1] Hodges SC, Mijumbi C, Okello M, McCormick BA, Walker IA, Wilson IH (2007). Anaesthesia services in developing countries: defining the problems. Anaesthesia.

[2] Gawande A (2012). Two Hundred Years of Surgery. N Engl J Med.

[3] Barash PG, Cullen BF, Stoelting RK (2001). Clinical anesthesia.

[4] Bigelow HJ (1846). Insensibility during Surgical Operations Produced by Inhalation. Boston Med Surg J.

[5] Bryan CS, Podolsky SH (2009). Dr. Holmes at 200—The Spirit of Skepticism. N Engl J Med.

[6] Holmes OW. “An introductory lecture, delivered at the Massachusetts Medical College” 1809–1894; http://collections.countway.harvard.edu/onview/items/show/6315. Accessed December 29, 2013 2013.

[7] Funk LM, Weiser TG, Berry WR, Lipsitz SR, Merry AF, Enright AC (2010). Global operating theatre distribution and pulse oximetry supply: an estimation from reported data. Lancet.

[8] Totonidis S (2005). A role for trichloroethylene in developing nation anaesthesia. Kathmandu Univ Med J (KUMJ).

[9] Bosenberg AT (2007). Pediatric anesthesia in developing countries. Curr Opin Anaesthesiol.

[10] van der Kleijn E (1989). Effects of zopiclone and temazepam on sleep, behaviour and mood during the day. Eur J Clin Pharmacol.

[11] Eghtesadi-Araghi P, Sohrabpour A, Vahedi H, Saberi-Firoozi M (2008). Halothane hepatitis in Iran: a review of 59 cases. World J Gastroenterol.

[12] Thomas GJ (1960). Fire and explosion hazards with flammable anesthetics and their control. J Natl Med Assoc.

